# Evaluation of Profile Changes in Class II Individuals Treated by Means of Herbst Miniscope Appliance

**DOI:** 10.3390/dj8010027

**Published:** 2020-03-20

**Authors:** Stefano Martina, Maria Luisa Di Stefano, Francesco Paolo Paduano, Domenico Aiello, Rosa Valletta, Sergio Paduano

**Affiliations:** 1 Department of Medicine, Surgery and Dentistry “Scuola Medica Salernitana”, University of Salerno, 84081 Baronissi SA, Italy; smartina@unisa.it; 2 Department of Health, University “Magna Graecia” of Catanzaro, 88100 Catanzaro CZ, Italy; marialuisadistefano77@gmail.com (M.L.D.S.); paduano@unicz.it (S.P.); 3 Camplus Humanitas University of Milan, 20090 Milano MI, Italy; paduano.francesco@yahoo.it; 4 Discipline of Orthodontics, Department of Neurosciences, Reproductive Sciences and Oral Sciences University of Naples “Federico II”, 80131 Napoli NA, Italy; valletta@unina.it

**Keywords:** soft tissues, class II malocclusion, functional appliance, Herbst Miniscope, functional treatment

## Abstract

Background: To evaluate the profile changes following orthopedic/orthodontic treatment with the Herbst Miniscope^®^ appliance in subjects affected with Class II malocclusion with mandibular retrusion. Methods: A total of 44 patients presenting a skeletal Angle Class II malocclusion (ANB > 4°) due to mandibular retrusion and a cervical maturation stage between CS2 and CS3 were included in the study. Of these 44 patients, 22 (mean age 11.9 ± 1.3, HBT group) were treated using the Herbst appliance, while 22 (mean age 10.6 ± 1.3, CTR group) were followed for a 12-month observational period. A cephalometric tracing was performed at the beginning of treatment (T0) and after 12 months (T1). Results: In both groups there was a significant advancement of soft tissue pogonion (HBT = 3.5 ± 3.0 mm, *p* < 0.001; CTR = 2.2 ± 2.9 mm, *p* < 0.001), but the difference between the two groups was not significant (*p* = 0.172). On the contrary, both groups had a significant advancement of the mandibular sulcus (HBT = 3.7 ± 2.8 mm, *p* < 0.001; CTR = 1.2 ± 2.2 mm, *p* < 0.001) and a lower lip protrusion (HBT = 3.45 ± 2.51 mm, *p* < 0.001; CTR = 1.7 ± 2.7 mm, *p* = 0.008), but in both cases the HBT group showed a statistically significant greater increase in sulcus protrusion (*p* = 0.002) and lower lip protrusion (*p* = 0.029) than controls. There were no statistically significant effects on the upper jaw. Conclusions: The Herbst appliance advanced the lower jaw soft tissues.

## 1. Introduction

Class II malocclusion is a very frequent orthodontic problem, affecting about 33% of the population [1], with a mandibular retrusion in almost 80% of patients [2]. Due to the difficulty in assessing this aspect using cephalometry, aesthetic evaluation is commonly used to diagnose retrusion of the mandible [3]. This is consistent with the attention paid to the correlation between an orthodontic treatment and an aesthetic result, intended not only to obtain a good occlusion but also and above all to achieve an optimal final aesthetic. Facial aesthetic improvement is the main reason why patients look for orthodontic treatments [4]. In addition, young patients’ parents want an enhancement of the facial, dental and dento-facial aesthetic of their kids [5]. It is an important goal for clinicians too, which is why they keep it in great consideration while planning treatments [6].

In order to influence growth of maxillary and mandibular bones, orthopedic-orthodontic treatments have been used by clinicians from years, but their efficacy has been widely debated [7,8]. In a systematic review [9] the authors concluded that, at this time, there is not enough scientific evidence in literature to recommend orthopedic treatment with functional appliances in patients with skeletal Class II. Since this lack of evidence is due to a low number and poor quality of primary studies, new trials should be performed. 

There are several studies that have described appliances able to determine increases in mandibular growth, thanks to a mandibular propulsion [10,11,12,13]. In subjects with mandibular retrusion near their growth peak, functional bite-jumping devices are advisable [10,11]. In subjects with Class II division 2 malocclusion, before using functional equipment, a preliminary phase to advance the upper retroclined incisors is required [14]. 

One possible treatment for Class II patients with mandibular retrusion is the Herbst appliance. Based on the “Bite Jumping” principle, this device acts by stimulating the condylar growth and remodeling of the glenoid cavity into a lower and forward position [15,16]. In this way, a correction of the dental relationships and an improvement of skeletal harmony are possible [17,18].

Although aesthetic improvement is the patient’s primary goal, few studies have investigated the perception of profile and soft tissue improvements resulting from functional therapy [19,20]. Only one retrospective study investigated the effects of the Herbst appliance on the soft tissue profile by comparing it with an activator [21].

Thus, the aim of this study is to evaluate profilometric changes in patients with skeletal Class II caused by mandibular retrusion, obtained with a simplified version of the Herbst appliance (Herbst Miniscope^®^). The null hypothesis was that the Herbst appliance did not change the soft tissue profile of the patients.

## 2. Materials and Methods 

### 2.1. Sample

All subjects gave their informed consent for inclusion before they participated in the study. The study was conducted in accordance with the Declaration of Helsinki, and the protocol was approved by the Ethics Committee of Catanzaro (381) on 19 December 2019.

The sample comprised patients selected from Magna Græcia University’s dental clinic and orthodontic private practice. 

The inclusion criteria of this study were the following:

- a full Class II molar relationship;
- ANB > 4°,
- Overjet > 6 mm,
- an age range of 9–15 years,
- good quality radiographs, and
- cervical vertebral maturation stage (CVMS)2 or >3 [22].

The exclusion criteria were the following:

- hyperdivergent subjects (mandibular plane angle equal to or greater than the normal value plus a standard deviation 26° ± 4°) [23],
- tooth agenesis,
- previous orthodontic treatment, and
- periodontal diseases.

The final sample consists of 44 patients (22 girls and 22 boys, mean age 11.6 ± 1.3 years):

- a total of 22 patients (14 girls, 8 boys, mean age 11.9 ± 1.3 years, HBT group) were treated with Herbst MiniScope^®^ appliance. Transversal discrepancies and/or crossbites, if present, were treated with orthopedic or orthodontic expansion of the palate before functional treatment [24]. The average age of therapy onset was 10 years, 8 months for females and 11 years, 11 months for male. After the orthopedic phase, the patients underwent orthodontic treatment with self-ligating brackets [25] and initial thermoelastic or superelastic archwires [26];
- the CTR group comprised 22 patients (14 boys, 8 girls; mean age 10.6 ± 1.3,). The CTR group followed the same inclusion and exclusion criteria as the treated group.

The cervical stage was assessed by one examiner on the T0 cephalogram according to the cervical vertebral maturation (CVM) method [22,27].

The cephalograms had a mean time interval T1-T0 of 12 months.

### 2.2. Cephalometric Analysis

The differences in the T1-T0 linear measurements were recorded according to Pancherz’s method [17,28].

One examiner trained in electronic cephalometric analysis performed all the cephalometric measurements using Dolphin Imaging 11.0 software (Chatsworth, CA, USA). The measuring points, reference points, and lines were defined following Pancherz’s method. Where there was a double projection of two points, the midpoint was used. For all the linear measurements, the OL and the OLp of the initial radiograph were used as a reference grid. The grid was then transferred from the T0 to the T1 radiograph by superimposing it onto the N-T point line, with the T point as the registering point. All the linear measurements were made parallel to the OL.

The profilometric changes obtained after the treatment period were evaluated by comparing the lateral teleradiography performed at the time T0, just before the start of treatment, with that at the time T1, 12 months after the start of treatment, when the functional appliance was removed. A cephalometric tracing of the CTR group was performed at the time T0 and after 12 months of observation (T1) without any orthodontic treatment. Once the cephalometric analyses were carried out, the traces were overlapped on/superimposed on the NSL plane (Nasion Sella line), the inclination of which does not change with age, to evaluate the changes in the profile in each patient [28]. 

Soft tissue evaluation was performed by evaluating linear and angular measurements.

According to Pancherz’s method [28] the occlusal plane (OL) and occlusal line perpendicular (OLp) were used as the reference planes. OL is the plane passing between the disto-buccal cusp of the first upper molar and the upper incisors. OLp is a line perpendicular to OL passing through Sella (S).

All the tracings were performed by one single blinded operator who trained in electronic cephalometric analysis and conducted all the cephalometric measurements using Dolphin Imaging 11.0 software (Chatsworth, CA, USA).

From the Pancherz cephalometric reference planes, normally used to evaluate skeletal and dental displacements, the distances of the soft tissue points examined were evaluated [29,30].

For each subject the Nasion (NA) and T points from the first head film tracing were transformed to the second tracing by using the structures of the anterior cranial base for orientation.

The films were superimposed with great care so that the distance from the sella turcica was the same on both head film tracings. 

#### 2.2.1. Reference Lines 

**Figure 1:**
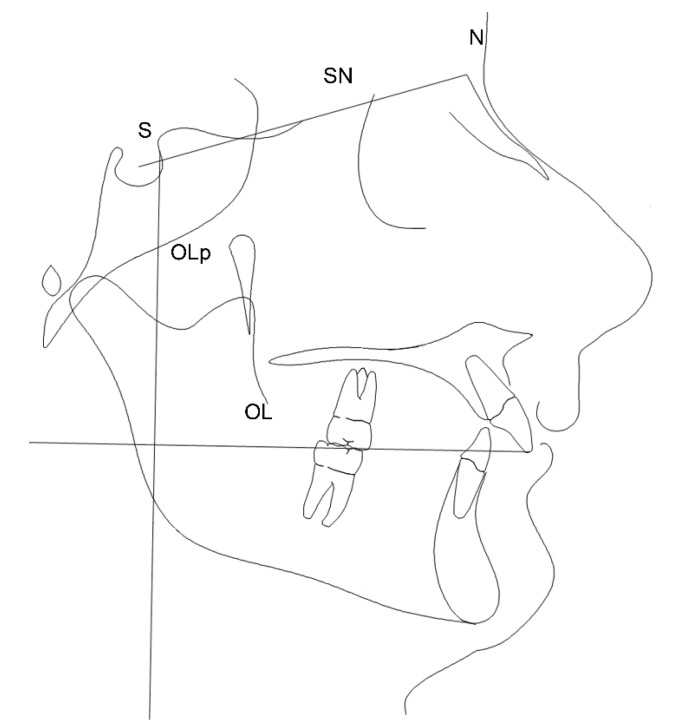
Cephalometric reference lines.

The reference lines used in the cephalometric analysis are shown in Figure 1.

All measurements were done parallel to OL.

For each patient, measurements were analyzed in the pre- and post-treatment periods (or after 12 months of observation) to evaluate profilometric changes:

- NSL: (nasion-sella line) S to N line used in cephalometric orientation
- OL: (occlusal line) line that goes from U1 to the distobuccal cusp of the first permanent upper molar. Used as a reference line in the detection of cephalometric measurements
- OLp: (perpendicular line to the occlusal plane) perpendicular to OL passing through T point. It represents the line from which the distances to the measuring points were evaluated.

#### 2.2.2. Measuring Points

**Figure 2:**
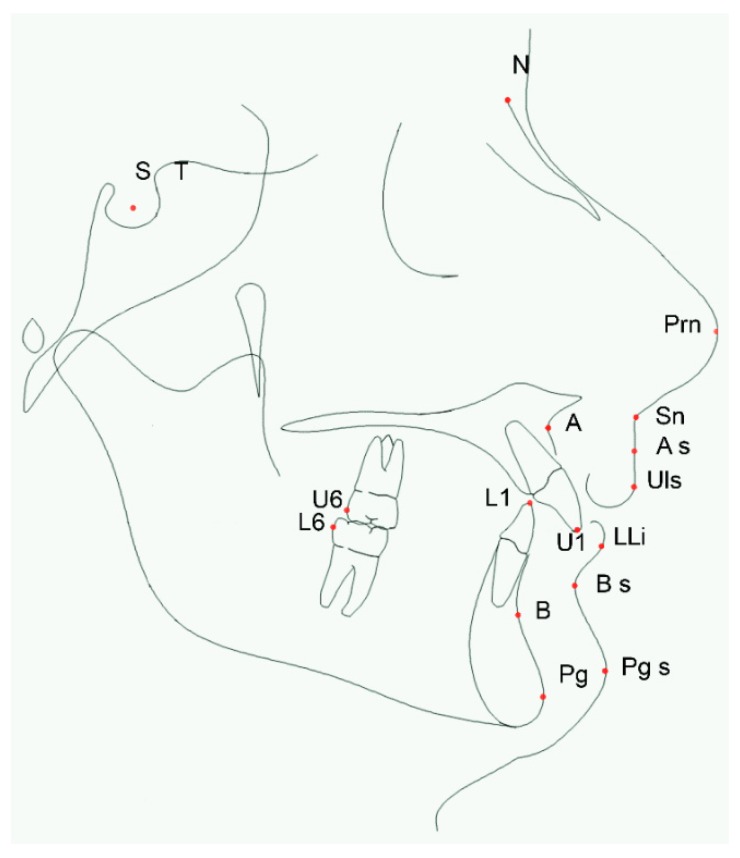
Cefalometric measuring points.

The measuring points are shown in Figure 2:

- S: (sella) point located at the center of the sella turcica
- T point
- N: (nasion) earlier point of the nasofrontal suture
- U1: (superior incision) lower point of the upper central incisors
- Prn: (nasal point), the most anterior point of the nasal apex
- ANS: (anterior nasal spine), most anterior point of maxillary bone
- A: (point A), posterior point of the anterior convexity of the maxilla
- A soft: (A soft point), maximum concavity of anterior lip superior point
- Sn: (nasolabial point), the point of maximum concavity of the nasolabial curvature
- ULs: (upper lip), the most forward point of the upper lip
- U6: (upper first molar), distal point of upper first molar
- L1: (lower incision) upper point of the lower central incisors
- LLi: (lower lip), most anterior point of the lower lip
- L6: (lower first molar), distal point of inferior first molar
- Sub: (inferior labial sulcus), the posterior point of the inferior labial sulcus
- B: (B point), maximum concavity of anterior mandibular bone
- Po: (pogonion) anterior point of the mandibular symphysis
- Pos: (soft tissue pogonion), most anterior point of the soft tissues of the lower jaw

#### 2.2.3. Linear Measuring Procedures

**Figure 3:**
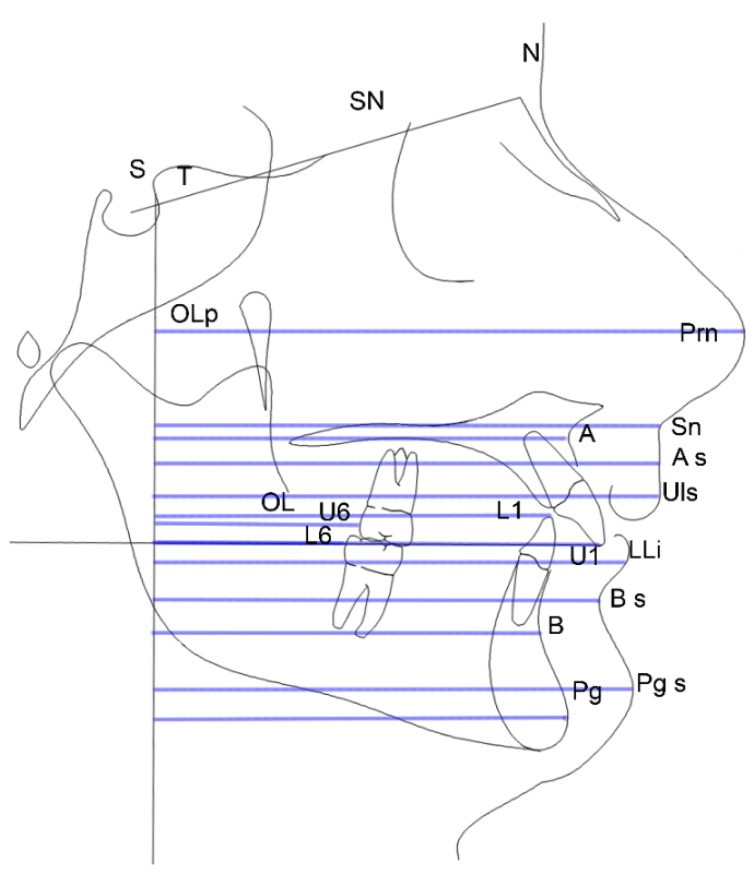
Linear measuring procedure.

Measurements were performed on lines parallel to the OL starting from the vertical reference line Olp and are shown in Figure 3:

- SN: sella-nasion line
- OLp-U1: line that evaluates the position of superior incisor compared to the perpendicular line to the occlusal line
- OLp-Prn: line that evaluates the nasal prominence compared to the perpendicular line to the occlusal plane (nasal growth)
- OLp-ANS: line that evaluates the anterior nasal spine point compared to the perpendicular line to the occlusal plane
- OLp-A: line that evaluates the maxillary’s position compared to the perpendicular line to the occlusal plane
- OLp-As: line that evaluates the position of A point of the soft tissue compared to the perpendicular line to the occlusal plane
- OLp-Sn: line that evaluates the position of the infranasal point compared to the perpendicular line to the occlusal plane (filtrum)
- OLp-ULs: line that evaluates the position of the upper lip compared to the perpendicular line to the occlusal plane
- OLp-U6: line that evaluates the position of upper first molar compared to the perpendicular line to the occlusal plane
- OLp-L1: line that evaluates the position of lower incisor compared to the perpendicular line to the occlusal plane
- OLp-LLi: line that evaluates the position of the lower lip compared to the perpendicular line to the occlusal plane
- OLp-L6: line that evaluates the position of the lower first molar compared to the perpendicular line to the occlusal plane
- OLp-B: line that evaluates the mandible’s position compared to the perpendicular line to the occlusal plane
- OLp-Sub: line that evaluates the position of the mandibular sulcus compared to the perpendicular line to the occlusal plane (concavity of the mandibular sulcus)
- OLp-Po: line that evaluates the position of the pogonion compared to the perpendicular line to the occlusal plane
- OLp-Pos: a line that evaluates the position of the soft tissue pogonion compared to the perpendicular line to the occlusal plane.

### 2.3. Statistical Analysis

The sample size calculation showed that 19 patients per group were needed to detect an increase in mandibular length ≥2.0 to achieve 80% of power according to previously estimates changes in mandibular length (Pg/OLp) [31].

Data were analysed by conventional descriptive statistics. A Shapiro-Wilk test was performed to evaluate wheter the samples were normally distributed.

Variables were analysed by means of paired t-test or within-group comparisons, while between-groups comparisons were performed by means of unpaired t-test. Absolute cephalometric changes were converted to relative changes over a 12-month period. The level of statistical significance was set at *p* < 0.05. All the analyses were performed with commercial software (SPSS version 22.0, SPSS IBM, New York, NY, USA).

## 3. Results

The values investigated were compared using the Student’s t-test. The mean and standard deviation (SD) were calculated for each variable, and the t-test was performed to compare the initial and final results of the period considered and to ascertain the differences between the groups. The magnitude of the methodological error for the linear measurements was calculated with the formula ± √Σ*d*2/2*n* where d is the difference between two measurements per pair and n is the number of double measurements. The methodological error does not exceed ±0.25 mm for each variable investigated. 

The results are shown in Table 1.

From the obtained data, a more anterior position from CTR group can be assessed:

- of 2 mm, on mean average, of the lower lip (OLp-LLI), *p* = 0.030
- of 4.3 mm, on mean average, of the sublabial sulcus (OLp-sublabial), *p* = 0.002.

## 4. Discussion

The aim of this article was to evaluate on cephalograms the sagittal changes of the profile in Herbst treatment. Cephalometric analysis helps the orthodontists evaluate their treatment results, taking into account profile changes. This evaluation is obviously limited to sagittal changes.

It must be remembered that the patients selected presented a skeletal Angle Class II malocclusion with mandibular retrusion and favorable functional treatment response. 

The results of this study support the hypothesis that functional appliances positively influence the profile of patients with Class II malocclusion. 

This is consistent with the results of other studies that were carried out with other functional appliances [32,33,34]. 

According to the literature, there is a moderate increase in nasal prominence, in the position of the filtrum and the upper lip, most likely ascribed to the patient’s growth and not to the action of the appliance. A significant increase in the position of the lower lip and pogonion, albeit partly due to patient growth, is undoubtedly favored by the mandibular propulsion mechanism resulting from the therapy with Herbst Miniscope^®^. This confirms the findings of previous studies on Herbst appliance [35,36]. 

Although the influence of the functional treatment on mandibular growth appeared limited, and although the change observed in mandibular position could be ascribed mainly to normal growth, a significant improvement in the patient’s profilometric characteristics was observed.

Above all, there was a noticeable advancement of the lower lip compared to the superior, limiting the gap existing before the therapy between the two structures. This advancement should be ascribed to a more protrusive position of the mandible determined by the Herbst appliance. The change in value of L1-LLi was due to a new muscle adaptation resulting from mandibular advancement. These results are similar to those of a recent study by Hourfar et al. [21] that retrospectively compared the profile improvements in patients treated with a Functional Mandibular Advancer and with a Herbst appliance, demonstrating that the Herbst appliance is more effective in modifying the soft tissue profile and advancing the lower lips. 

The results are also in accordance with two studies [20,37] that investigated the perception of facial attractiveness assessed by orthodontists, general pratictioners and laypersons after Herbst appliance treatment. Another study [38] reported no significant improvement in perceived facial changes after Class II treatment with functional appliances and fixed therapy, but the patients were not treated with the Herbst appliance.

## 5. Conclusions

The profile changes following orthopedic/orthodontic treatment with Herbst MiniScope^®^ fixed appliance in subjects affected with class II malocclusion with mandibular retrusion were evaluated cephalometrically. The material consisted of 44 Class II subjects with mandibular retrusion, 22 treated with Herbst appliance and 22 in the control group. Lateral roentgenograms in centric occlusion were analyzed before and after treatment. The original occlusal line (OL) and the occlusal line perpendicular (OLp) through sella were used as reference.

In conclusion, data from the study shows a significant improvement of the patient’s profilometric characteristics, resulting in an excellent correction of class II. It is advisable to carry out the correction of sagittal discrepancies as early as possible.

The dentoalveolar compensation determined by the Herbst device in some subjects is able to determine an improvement of the profile even in absence of significant skeletal changes. Therefore, the new Herbst Miniscope^®^ equipment is definitely valid for improving the profile of patients with class II malocclusion caused by mandibular retrusion, favoring a progression of the lips and the chin. Further studies are needed to evaluate the profilometric changes in the long term determined by the appliance.

## Figures and Tables

**Table 1:** Linear cephalometric measurement before (T0) and after (T1) the treatment/observation period. Data are reported as mean and standard deviation for patients treated with Herbst appliance (HBT) and controls (CTR) groups. Absolute cephalometric changes (T1–T0) are converted to relative changes over a 12-month period. The paired and unpaired t-test were used for the statistical analysis. The significance level was set at *p* < 0.05. The bold type indicates statistically significant differences between groups.

	CTR							HBT							
	T0		T1			DIFF		T0		T1			DIFF		HBT vs. CTR
	Mean	SD	Mean	SD	*p*	Mean	SD	Mean	SD	Mean	SD	*p*	Mean	SD	*p*
*Soft tissue points*															
PRN	85.4	3.6	87.6	3.7	0.000	2.3	2.3	87.0	4.5	89.7	4.5	0.000	2.5	1.5	0.702
A_S	78.3	4.4	79.7	3.8	0.026	1.3	2.8	78.0	4.7	80.3	4.3	0.000	2.2	1.9	0.230
SN	77.0	4.0	78.2	3.5	0.005	1.2	2.0	77.8	4.3	80.0	3.9	0.000	2.1	1.7	0.128
ULS	80.7	4.7	82.5	3.5	0.016	1.8	3.6	81.6	4.9	83.8	4.4	0.000	2.1	2.1	0.724
LLI	78.6	5.5	80.7	4.4	0.008	2.1	3.4	78.6	4.2	82.8	4.1	0.000	4.3	3.1	0.030
Sub	71.9	5.0	73.4	4.6	0.017	1.5	2.8	72.2	3.9	76.5	4.4	0.000	4.6	3.5	0.002
PG_S	75.1	5.4	77.7	4.7	0.001	2.8	3.7	76.4	5.0	80.5	5.1	0.000	4.4	3.8	0.173
*Hard tissue points*															
A	64.3	3.4	65.2	3.6	0.063	0.9	2.4	65.0	3.5	66.4	3.3	0.001	1.5	1.8	0.371
B	62.5	4.6	64.1	4.2	0.009	1.7	2.9	63.2	3.8	67.0	3.9	0.000	4.0	3.5	0.018
PG	65.0	5.1	67.5	4.7	0.006	2.7	4.7	66.3	4.5	70.4	4.4	0.000	4.3	4.0	0.247
U1	72.1	4.8	73.5	3.8	0.011	1.5	2.7	71.9	3.3	73.7	3.7	0.000	1.8	1.7	0.693
L1	65.4	4.6	66.8	4.1	0.007	1.4	2.5	64.7	3.6	70.3	3.8	0.000	5.7	3.5	0.000
U6	32.9	3.6	34.8	3.4	0.003	1.9	2.8	33.8	3.6	33.3	2.9	0.317	−0.4	2.0	0.003
L6	31.3	3.7	33.4	3.5	0.000	2.3	2.5	32.1	3.9	37.0	3.6	0.000	5.0	3.2	0.003
